# Supplementary material for: Transgenerational effects on development following microplastic exposure in Drosophila melanogaster
Source: PeerJ. 2021 May 7;9:e11369. doi: 10.7717/peerj.11369 (PMC8109007; doi:10.7717/peerj.11369)
Supplement: Supplemental Information 1 [file peerj-09-11369-s001.docx]

| Laboratory Normal Cornmeal Diet | | |  | |  |
| --- | --- | --- | --- | --- | --- |
|  | **Cornmeal** | **Dextrose (g)** | **Yeast** | **Agar** | **Totals** |
|  | **88** | **88** | **19** | **11** | **206** |
| Kcal | **325.6** | **321.2** | **67.45** | **2.86** | **717.11** |
| Fat (g) | **2.464** | **0.088** | **1.52** | **0** | **4.072** |
| Carbohydrate (g) | **69.52** | **83.6** | **3.61** | **0.77** | **157.5** |
| Protein (g) | **6.16** | **0** | **8.265** | **0.055** | **14.48** |
| Salt (g) | **0** | **0** | **0.024795** | **0** | **0.024795** |
| Sugars (g) | **1.408** | **83.6** | **2.66** | **0.033** | **87.701** |
