# Supplementary material for: Transgenerational effects on development following microplastic exposure in Drosophila melanogaster
Source: PeerJ. 2021 May 7;9:e11369. doi: 10.7717/peerj.11369 (PMC8109007; doi:10.7717/peerj.11369)
Supplement: Supplemental Information 2 [file peerj-09-11369-s002.docx]

|  | F1 | | | F2 | | | |
| --- | --- | --- | --- | --- | --- | --- | --- |
|  | Control | PE | PVC | Control | PE | PVC1 | PVC4 |
| Males | 407 | 312 | 301 | 231 | 399 | 235 | 383 |
| Females | 403 | 360 | 376 | 228 | 410 | 234 | 361 |
| Total | 810 | 672 | 677 | 459 | 809 | 469 | 744 |
| % males | 50.25 | 46.43 | 44.46 | 50.33 | 49.32 | 50.11 | 51.48 |
